# EdgeTwin-DRL: Real-Time Counter-UAS Detection and Response Optimization Using Edge-Assisted Digital Twins and Multi-Agent Deep Reinforcement Learning

**DOI:** 10.3390/s26175632

**Published:** 2026-09-04

**Authors:** Abdulrahman K. Alnaim, Ahmed M. Alwakeel

**Affiliations:** 1 Department of Management Information Systems, College of Business, King Faisal University, Alahsa 31982, Saudi Arabia; 2 Faculty of Computers & Information Technology, University of Tabuk, Tabuk 71491, Saudi Arabia; aalwakeel@ut.edu.sa

**Keywords:** digital twin, drone intrusion detection, deep reinforcement learning, edge computing, counter-drone systems, airspace security, multi-agent reinforcement learning, real-time response optimization

## Abstract

The time available for a counter-drone system to detect an unauthorized aircraft and determine an appropriate response can be limited. Although cloud processing remains useful for storage and offline analysis, communication delays may constrain its use in time-critical decision loops. This paper proposes EdgeTwin-DRL, an edge-assisted digital twin framework that integrates multimodal sensing and multi-agent deep reinforcement learning (DRL) for counter-drone detection and response optimization. The digital twin maintains a synchronized representation of the protected airspace using radar, electro-optical/infrared (EO/IR), radio-frequency (RF), and acoustic observations. This synchronized state is used by cooperative DRL actors to adjust computational-resource allocation, detection sensitivity, and candidate countermeasures, while a model-based forward-evaluation assesses proposed responses before they are passed to the simulated response pathway. The framework is evaluated in a simulation testbed in which the RF sensing models are calibrated and independently validated using publicly available datasets, while the remaining sensing components are parameterized using published experimental measurements. Within this calibrated simulation environment, EdgeTwin-DRL achieved a false-positive rate of 1.4% and reduced mean detection-to-response latency by up to 72% relative to the Cloud-DRL baseline and by 26% relative to the MAPPO baseline without calibrated, environment-dependent sensing under the communication and computational assumptions used in the simulator. The evaluation was conducted across modeled urban, suburban, and open-field conditions. These results demonstrate the comparative performance of the proposed architecture within the simulated environment and motivate further investigation of edge-assisted digital twins for counter-drone decision support. Hardware-in-the-loop and controlled field validation are required before operational deployment.

## 1. Introduction

The commercial unmanned aerial vehicle (UAV) has become a cost-effective, capable and easily deployable weapon. Small drones are also a threat to facilities like refineries, substations and airports because of these benefits. In the last couple of years, there have been several examples of the disruption of energy operations with modified commercial drones and/or the creation of significant safety hazards with relatively low-cost aircraft that are classified as drones [1,2] Defenders struggle with drones because they could carry a payload and because their operational characteristics present significant challenges to conventional detection and counter-UAV systems [3]. Small UAVs typically navigate at altitudes low enough to ensure they are not detected by any conventional perimeter security systems, have a small radar cross-section, and are able to approach through areas designed for conventional perimeter security.

No one sensor is considered to be definitive in this scheme. The range and motion data can be provided by radar, a control or data link can be added by RF monitoring, visual or thermal confirmation is added by EO/IR and a nearby rotor signature can be added by acoustic sensing [4]. The usefulness of combining them is not academic—clutter can cause tracking errors on a radar, radio silence will make it harder to hear because they have no RF cue, equipment can mask acoustic characteristics and camera performance varies depending on illumination and visibility [5]. Intrusion detection must then make a response decision in a timely manner once a possible intrusion has been detected. There are many examples of how a small UAV traveling at a speed of 60 km/h can consume a considerable portion of the available warning time while reviewing the uncertain evidence.

The immediate decision loop has been deployed in a cloud service, which is remote in this case. While a cloud server could be beneficial for storage and offline processing, published surveillance and edge compressing measurements indicate that the latency introduced by offloading varies between hundreds of milliseconds and seconds depending on the network conditions [6]. In this application, this delay is not the only networking overhead; it is also a part of the response time. It also shows reliance on the outside link during an intrusion. The proposed architecture thus implements time-sensitive inference and coordination in close proximity to the sensors and delays the “slower” tasks to the cloud [7].

MEC is responsible for the deployment of that option [8]. This will enable edge nodes near the sensing perimeter to process the incoming observations, update tracks and send a response recommendation without having to upload the raw streams. The trade-off is that edge hardware has a limited computational budget. If there is a quiet period, then there is a rich supply of budget, but if there are a number of unclear tracks, then there may be competing requirements for preprocessing, fusion and classification. Then, the response is selected, the track is confirmed and resources are allocated to form a single connected decision-making task.

The decision problem is arranged by using a digital twin. In this study, the twin is not just a 3-D representation of the facility. It is a time-aligned state model, as measurements of various sensors are related to the respective tracks [9]. The same model can be used to predict a short-term result of a proposed countermeasure. It also offers a space for training in a controlled setting where rare or risky cases of intrusions can be repeated without having to risk equipment or people [10]. The twin has therefore two functions in our task: maintaining the state used when making the decision and providing the scenarios for training the policy.

The controller is called DRL, because the controller has to make a series of interconnected decisions, rather than “make one label”. The action that is beneficial in one instant could include spending more processing time on an uncertain track, increasing or decreasing the sensitivities of the detector, or determining a response when the evidence has reached a sufficient level. When real intrusion events are labeled, it is difficult to learn these trade-offs, but simulated interaction offers a means for doing so [11]. Since the sensing and computation are distributed among multiple edge nodes, a multi-agent formulation is used, where each node acts on a local level, but is trained towards a common goal [12]. Previous DRL counter-drone research shows simulated interception policies [13]; the previous decisions of “when” and “where” to detect and allocate an interception are considered, which ultimately result in a recommendation for intervention. More challenging evasive behaviors may also involve highly nonlinear or chaos-based path-planning, in which sensitivity to initial conditions can produce non-repeating and difficult-to-predict UAV trajectories. Such behaviors could impose additional demands on track prediction, sensor coordination, and response decision-making beyond those associated with conventional straight, loitering, or zigzag trajectories.

This leaves a particular gap. In sensor-fusion studies, the detection accuracy is usually measured without taking into account what actions should be taken on a detected threat when the edge-compute budget is limited. Studies that are response-focused may take for granted that the information about the target state is reliable. Digital-twin and MEC studies, however, have primarily focused on industrial monitoring or aiding network optimization, not on protection against counter-criminal activity such as a drone attack. Previous publications have presented cooperative DRL-based multi-objective (detection, resource allocation, and response) decision-making using an edge-hosted airspace twin.

EdgeTwin-DRL does not introduce a new reinforcement-learning algorithm or a new general-purpose digital twin modeling theory. Instead, its contribution lies in the formulation and architectural integration of four functions that are usually treated separately in counter-UAS and edge-intelligence research: heterogeneous intrusion sensing, edge-resource allocation, synchronized airspace-state estimation, and response recommendation. The protected airspace is represented as a continuously updated operational state shared by the sensing and decision layers, while cooperative edge agents jointly optimize computational allocation, detection sensitivity, and response selection. Thus, the methodological contribution of EdgeTwin-DRL is the construction and evaluation of an integrated counter-UAS decision loop rather than a modification of the underlying MAPPO algorithm.

(1) This paper proposed a four-layer architecture, which synchronously optimizes both detection confidence and compute allocation to a multi-agent DRL controller, as well as response selection, with a distributed edge processing layer coupled with a digital twin of protected airspace.
(2) The digital twin is used in two ways: it is a synchronized operational model for multi-modal sensor fusion and it is a simulation environment for the training and pre-deployment of policies and checking the countermeasures.
(3) This paper represents the decision problem as a cooperative multi-agent Markov decision process (MAMDP) and it is solved with PPO with decentralized actors and a centralized critic adapted to the distributed edge nodes.
(4) An assessment of the framework was performed in a simulated testbed which emulates the edge tier and integrates radar, RF, EO/IR and acoustic observations. In the simulated environment, EdgeTwin-DRL reduces detection-to-response latency by up to 72% when compared to a cloud-centralized baseline with a 1.4% false-positive rate across three scenarios—urban, suburban, and open field.

Section 2 summarizes the work that led to this design, and explains the remaining gap. The model facility, sensors, edge tier and digital twin are described in Section 3. The learning formulation is given in Section 4. The protocol for conducting the simulations, and the comparisons that were made, are described in Section 5 and Section 6. The paper ends with Conclusions and Future Works in Section 7.

## 2. Related Work

Our design is directly inspired by three distinct lines of research: counter-drone sensing and response, digital twins for security monitoring, and learning-based resource management at the edge. While this paper addresses these domains separately to highlight their individual contributions, this paper focuses on how they dynamically interact within a single counter-drone decision cycle.

### 2.1. Counter-Drone Detection Technologies

As a rule, a practical installation of a counter-drone system will use multiple sensor installations, given that no one sensor measurement is reliable in every scenario. Different sensors (radar, RF monitoring, EO/IR cameras and acoustic arrays) observe different properties of the same target, and thus they all do not succeed.

Although the use of daylight was supplanted by infrared video surveillance, radar remains the obvious solution for area surveillance. It is not a simple solution for small UAVs. They have a small radar cross-section and they fly in the area of the picture with the greatest ground clutter. These constraints are discussed in the context of counter-UAS by Brown [5]. The rotor micro-Doppler effect can still be used to aid in discrimination in millimeter-wave FMCW systems [14], but structures, reflections and obstructed views are still relevant in site deployment.

A different aspect of the problem is addressed by RF sensing: emissions from the drone to its controller. Al-Sa’d et al. [15] were able to identify drone models and operating states from controlled data with the help of RF features. However, the sensor does not contribute much when the aircraft is at a pre-programmed track or the aircraft does not have a detectable transmission. The acoustic sense is a local cue, independent of the others. Ding et al. [16] fused acoustic and optical tracking, and mentioned that the noise in the environment also brought a difficulty.

These complementary weaknesses are the basis of sensor fusion. Frid et al. [17] used both RF and acoustic features and Lee et al. [18] employed a convolutional model to fuse radar and camera observations. The two types of work are beneficial in terms of the reliability of detection but are only effective in the detection or classification stage. They do not decide on the number of limited edge resources to distribute during an intrusion, nor do they decide on the response to be selected after combining several sensors.

In a 3D simulation, the generation of SHAP-explanations for the learned decisions was explored in later studies [4], while Çetin et al. [13] trained and compared a DQN-based interception policy in a three-dimensional simulation. These studies start with an objective to shoot down. Our problem begins earlier: it contains the uncertain multi-sensor information, has limited processing resources at the edge, and offers an outcome (recommendation to respond) based on an agreed airspace state.

### 2.2. Digital Twins for Security and Intrusion Detection

Digital twins were originally physical assets and processes but in the context of security, the benefits are maintaining a simulated environment for testing alternatives and comparing a system as-measured with one as-expected [9]. Jeremiah et al. [19] also point out that a twin is not necessarily secure: synchronization channels, model fidelity, and data integrity are also security issues.

Multiple intrusion-detection investigations are based on the principle of deviation from a real process and its twin. Such an approach has been developed by Balta et al. [20] for the production of cyber-physical systems. El-Hajj et al. [21] discussed the application of a digital twin in the field of smart-city IoT security; Krishnaveni et al. [22] introduced a digital twin and software-defined networking to an industrial ID. While these studies confirm the possibility of using a twin for context-aware monitoring, the attack surfaces and how to respond to them are different in low-altitude airspace.

The use of digital twins with UAVs has largely focused on communication, data collection, and/or fleet operations. Yigit et al. [23] proposed TwinPort, which integrates 5G drone-assisted data collection with a Digital Twin for smart seaports, while other studies have considered Digital Twin applications for autonomous core-network security [24] and autonomous drone systems [25]. The approach to model features is different from the approach in prior work in that the present work models the protected airspace, not just the intruding drone fleet, and then relates sensor fusion, policy training and response evaluation to that model.

### 2.3. Deep Reinforcement Learning for Edge Computing and Resource Management

The decision on computation and communication can vary based on the demand, the quality of the links and device capabilities, and that is why DRL is so widely used in edge-resource studies. In environments where it is hard to exhaustively optimize (e.g., online), the underlying MEC offloading problem and adaptive decision policies are summarized by Mach and Becvar [8].

In the single-agent context, Xiong et al. [26] used deep Q-learning for offloading and resource allocation and Chen et al. [12] studied the application of DRL in multi-UAV-assisted MEC networks. If the entire action space can be represented by a central controller, then such formulations can be useful. But in a distributed sensing system, the size of the joint state and action space increases rapidly with the addition of new nodes and sensor tasks.

When local decisions need to be made by each agent with respect to a specific time, but the agent should not optimize in isolation, multi-agent DRL is applicable. Yu et al. [27] introduced MAPPO, which is a centralized training with decentralized execution (CTDE). Training can take advantage of the joint state, while the deployed actors only need to rely on their local observations. Seid et al. [28] and Suzuki et al. [29] employ slightly similar task offloading and cooperative edge computation techniques. While they are used in a different application, the coordination structure is adequate for the problem of distributed sensing studied here.

Several recent research works have already correlated digital twins to reinforcement learning at the edge. Zhao et al. [30] apply a twin in a vehicular offloading problem, and Lu et al. [10] study digital-twin-assisted edge association as well as federated learning and blockchain. The listed studies demonstrate that a twin can assist in learning decisions in networks with limited resources. They do not involve protected airspace modeling, nor combine the detection of counter-drones with a recommendation for action.

### 2.4. Summary and Research Gap

Table 1 matches typical studies to their functionality in the proposed system. While there are existing works that cover one or more of the capabilities mentioned above, no work is listed that covers all five capabilities evaluated here.

The gap is easier to see when displayed in tabular form as in Table 1. By conducting counter-drone sensing studies, one can add more evidence to the detection, and by conducting interception studies, one can start when the target state has been successfully assumed. Typically, digital-twin security focuses on a cyber-physical or networked system, and not on an airspace track picture. Finally, multi-agent edge studies do not take into account the decision on a countermeasure. EdgeTwin-DRL examines the consequences of having these functions in a single simulated operating loop.

## 3. System Model

**Figure 1:**
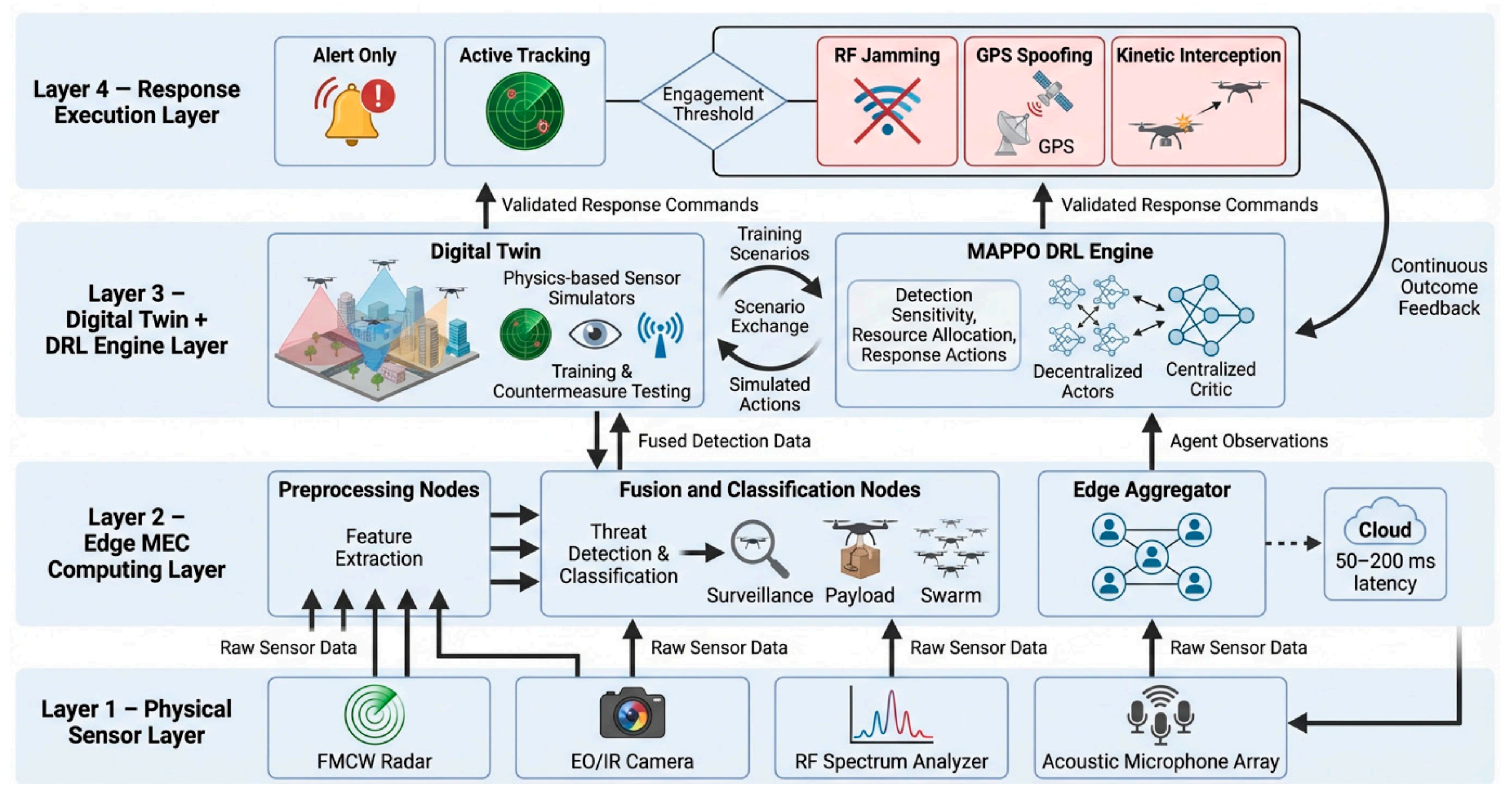
EdgeTwin-DRL system architecture.

This section specifies how the EdgeTwin-DRL is modeled in its operating environment. The design consists of four layers: physical sensing, edge computation, digital twin, and response execution, which is linked to the DRL controller. The information path from the observations from a sensor to a validated response command is illustrated in Figure 1.

### 3.1. Deployment Scenario and Threat Model

A sensitive facility is an oil refinery, power station or airport, where a geofence is monitored. When an aircraft enters this area without permission, it is considered a ‘candidate threat’ and monitored until it is cleared or a response is recommended. The protected area modeled covers an altitude range from ground level to 500 m, and laterally from the facility center to 2–5 km, similar to the assumptions for restricted areas adopted in counter-UAS studies [31].

The scenario generator is used to represent an adversary that comprises one or more commercial-class drone(s) less than 25 kg. There are three categories because the different ones require different things of the controller. Category I represents a slower surveillance aircraft. Category II represents a payload-carrying drone that takes a fairly direct route towards the protected asset. C3 is a set of aircraft that come from multiple directions at the same time, which necessitates multiple tracking and response requirements. In these classes, the altitudes, RF operation and path regularity are set to allow for training to not be limited to continuously transmitting drones on simple straight-line approaches.

The threat model also permits partial observability across sensing modalities. A UAV may therefore provide weak evidence in one or more sensing channels because of a reduced radar cross-section, RF silence, poor visibility, environmental clutter, or a low signal-to-noise ratio. These conditions are represented through the randomized radar cross-section, RF-emission availability, sensor-noise, detection-range, clutter, and visibility parameters used by the simulator. The framework consequently does not require simultaneous confirmation from all four modalities; the available sensor evidence is combined through the track-level fusion and digital twin state-updating process.

### 3.2. Heterogeneous Sensor Architecture

The sensor physical-layer model contains four types of sensors. This is because of the difference between the strengths of the sensors, meaning the same track might not have a strong signal in one channel but may have a reasonably good one in another. The fusion stage can then be assured of having sufficient observations to confirm a target from a single stream.

Radar Units: The long-range part of the model comprises an X-band FMCW radar of 9.0–10.0 GHz with a 5 km range. It generates range-Doppler observations with 20 Hz, and the micro-Doppler information of the rotor is extracted at the initial stage to discriminate the rotor [14]. Overlapping views are provided around the perimeter by multiple assumed placements. For every radar observation, the range, azimuth, elevation and Doppler estimates are sent to the preprocessing node.

Electro-Optical and Infrared (EO/IR) Cameras: The visual model includes co-aligned visible and long-wave infrared (LWIR) channels on the Electro-Optical and InfraRed (EO/IR) camera. The visible stream is set to 1920 × 1080 at 30 frames per second and the infrared stream is the thermal contrast from motors and batteries. If a radar track is available, the digital twin provides a pointing region prediction which can be used by the EO/IR model to analyze a directed region, instead of looking through an entire scene without a cue.

RF Spectrum Analyzers: These receivers are used for monitoring the 400 MHz through the 6 GHz band for control-link and video-downlink emissions. For the available emissions, a classifier, trained from RF fingerprint data, guesses the platform family and communication protocol [15]. The separated receivers can also be used for a time-difference-of-arrival estimation of the controller’s location. This stream is explicitly set up to be a supporting piece of evidence; an independent or RF-less aircraft might not appear on this stream.

Acoustic Microphone Arrays: A 16-element circular array is modeled close to the ground and provides a short-range confirmation and bearing estimate. The beamforming stage will highlight the frequencies of the blade-pass and its harmonics and project them in relation to the ambient background [16]. However when a low-altitude target is RF-silent, this channel can be helpful, but its “effect would probably get worse with strong industrial/urban noise”.

All sensor observations are timestamped before entering the emulated edge tier. The digital twin then aligns them within a common spatial and temporal reference, associates them with existing tracks, and updates the fused track state supplied to the decision-making framework.

#### Multimodal Observation Alignment and Fusion

The four sensing modalities are combined using track-level feature fusion rather than raw-data early fusion. Each sensor first performs its modality-specific observation process, after which its output is represented by a common track-oriented observation containing the available kinematic information, detection or classification confidence, timestamp, and sensor identifier. Because radar, EO/IR, RF, and acoustic observations are generated at different rates and may not provide identical feature types, missing modality-specific attributes are not directly concatenated at the raw-signal level.

Before fusion, observations are temporally aligned according to their timestamps, transformed into the common airspace coordinate frame, and associated with an existing track using spatial and temporal consistency. For a track i at time t, let $z_{i,t}^{\left(m\right)}$ denote the aligned observation supplied by modality m∈ {Radar, EO/IR, RF, Acoustic}, and let $c_{i,t}^{\left(m\right)}\;\in [0,\;1]$ denote its associated confidence. The fused track representation can be expressed as

(1) $$\begin{array}{c} z_{i,t}^{fuse}=F\left(\left\{\left(z_{i,t}^{\left(m\right)},\;c_{i,t}^{\left(m\right)}\right)\right\}\;m\in M_{i,t}\right) \end{array}$$

where $M_{i,t}$ is the set of sensing modalities providing a valid observation for track i at time t, and F(·) denotes the confidence-aware track-level fusion operation. The resulting fused representation updates the persistent track state maintained by the digital twin, including position, velocity, heading, confidence, and threat score. When a modality does not provide a valid observation at a given update, the remaining available modalities continue to contribute to the track representation rather than requiring a complete four-sensor observation.

This design corresponds to feature/track-level multimodal fusion: modality-specific measurements are processed independently and aligned before being integrated into the common digital twin track representation. It therefore differs from raw-data early fusion and does not employ a learned attention-based fusion network. The fused track state is subsequently used to construct the local observations supplied to the edge agents, as is described further in Section 4.

### 3.3. Edge Computing Infrastructure

The edge tier consists of N compute nodes connected through a dedicated low-latency Ethernet network distributed around the sensor perimeter. The platform to be used is an NVIDIA Jetson module based on an NVIDIA Orin class GPU which is reported to have up to 275 TOPS for AI inference (Reference [32]). However, no physical Jetson device was used in the reported experiments. On the other hand, the capability and queue limits of the edge nodes are simulated, as explained in Section 5.1.

Preprocessing Nodes are responsible for pre-processing of the sensor data: radar range-Doppler processing, EO/IR object detection/tracking, RF spectral feature extraction, and acoustic beamforming. These workloads are parallel workloads and the policy assigns the workloads to the GPU resources.

Fusion and Classification Nodes fuse together features from the modalities available, match observations to tracks and provide an initial threat class estimation. The digital twin provides the shared coordinate and timing reference that is required to combine measurements that come in at different rates.

The Edge Aggregator is responsible for orchestration of the multi-agent learning architecture. It is used as the centralized critic to evaluate joint actions during training with the global state. In deployment, it collates the overall observations required for coordination and is able to send out policy updates as needed. It can also interface with a cloud service for less time-sensitive tasks, like log storage or model evaluation offline processing.

Time-critical inference and response selection are therefore performed within the modeled edge tier, while cloud resources are reserved for non-time-critical functions such as log storage and offline model evaluation. Cloud resources are not assumed to be present during an intrusion, but are available as an option. This separation reduces the impact of external-delay or interruption to the immediate response loop [6].

### 3.4. Digital Twin Design

EdgeTwin-DRL employs digital twin as an active component of the counter-UAS decision pipeline rather than as a visualization-only representation. In the proposed architecture, the twin has three distinct functions: (1) maintaining a persistent and synchronized representation of the protected airspace and edge-resource state, (2) providing the simulation environment used for policy training, and (3) evaluating candidate response actions before they are released through the simulated response pathway. These functions should be distinguished from one another because the operational-state function of the twin is different from its use as a training simulator. In the present study, both functions are implemented within the simulation environment; connection to physical sensors, edge devices, and response systems remains a future deployment stage.

#### 3.4.1. Online State Synchronization

The operational state maintained by the digital twin includes the active track list, facility geometry, terrain, geofence boundaries, selected environmental variables, edge-node resource utilization, and availability of response resources. For each active track, the maintained state includes estimated position, velocity, heading, detection confidence, and threat score. These variables form the shared airspace context from which the local observations provided to the edge agents are constructed.

During simulated operation, each incoming sensor observation is associated with a timestamp, sensor identifier, measurement confidence, and track hypothesis. Because radar, EO/IR, RF, and acoustic sensors operate at different sampling rates, observations are first aligned temporally and transformed into a common spatial reference before being associated with existing tracks. The paper describes the corresponding multimodal alignment and track-level fusion procedure in the Section entitled Multimodal Observation Alignment and Fusion. Track association is based on spatial and temporal consistency with the current twin state. The twin therefore maintains a persistent state that is incrementally updated as new measurements arrive rather than reconstructing the complete airspace representation independently at every policy step. This persistent representation preserves track continuity across successive observations and allows for temporary missed detections to be handled through the maintained track state rather than treating each measurement independently. However, persistent sensor biases are not explicitly estimated as latent state variables in the present implementation.

Two synchronization rates are used. Fast-changing variables, including track observations, threat confidence, and edge-compute utilization, are refreshed at approximately 20–50 Hz, whereas slower contextual variables, such as weather and facility status, are updated at approximately 1–5 Hz. This multi-rate update mechanism keeps time-critical information responsive without requiring all contextual variables to be recomputed at the highest sensor-update frequency.

The synchronized state is subsequently supplied to the edge actors through their local observation vectors. Actions selected by the actors, such as changes in computational allocation, detection sensitivity, or proposed response, modify the simulated operating state and influence the observations generated at subsequent decision steps. Thus, the present implementation contains a closed digital information loop between observation, twin-state updating, policy decision, and simulated system response.

It is important to distinguish this simulated bidirectional interaction from physical deployment. In an operational implementation, the input side of the loop would receive live sensor and edge-device telemetry, while validated outputs would be transferred to a human-supervised response interface or authorized execution system. Such physical bidirectional integration is not evaluated in the present study.

#### 3.4.2. Simulation and Policy Training

The digital twin also provides the environment in which the multi-agent policy is trained. During training, episodes are generated with varying numbers of UAVs, trajectory patterns, speeds, RF operating modes, weather conditions, visibility levels, clutter conditions, and sensor uncertainty. The sensor components reproduce the modeled range, update frequency, environmental dependence, and noise characteristics of the radar, EO/IR, RF, and acoustic sensing modalities. The parameters of these observation models are calibrated prior to policy evaluation using the publicly available RF datasets and the published sensor measurements described in Section 5.2. This calibration should not be interpreted as continuous real-time recalibration from deployed physical sensors; rather, it establishes the observation-model parameters used in the present simulation. During each simulated episode, the digital twin continuously updates its operational state as new simulated observations become available.

Using the twin as the training environment allows the policy to encounter rare, coordinated, and potentially hazardous intrusion scenarios repeatedly without requiring physical UAV incursions or active countermeasure trials. Domain randomization is further applied, as described in Section 4.6, to reduce dependence on a single set of environmental and sensor parameters. Nevertheless, the resulting policy remains a simulation-trained policy and requires hardware-in-the-loop and physical-site validation before operational deployment.

#### 3.4.3. Candidate-Response Evaluation and Decision Feedback

The third digital twin function is the evaluation of candidate response actions before they are released through the simulated response pathway. Once the fused threat confidence exceeds the engagement threshold, the multi-agent controller may propose a response such as continued tracking and alerting, RF jamming, GPS-related disruption, kinetic interception, or a combined action. The proposed action is not executed immediately. Instead, the current synchronized twin state is used to conduct a short forward-evaluation of its expected effect.

For a candidate response $a^{resp}$ applied to the current digital twin state s, the response-evaluation model estimates two quantities: the probability that the proposed action successfully neutralizes or diverts the simulated threat, $P\left(success,|a^{resp}s\right)$, and the associated collateral cost, $C\left(collateral,|a^{resp}s\right)$. The response-success estimate is generated by the simulator from the candidate response together with the current target state, including position, velocity, heading, threat level, response-resource availability, and the modeled operating conditions of the selected response. The collateral-cost term represents the modeled penalty associated with applying that response under the same state, including possible effects outside the intended target or permitted response conditions. These two model-derived quantities are then used in the response-effectiveness reward defined in Equation (8). They are simulation outputs used to rank or reject candidate actions and should not be interpreted as empirically measured probabilities or costs from physical counter-UAS engagements.

For each candidate response, the simulation evaluates its expected effectiveness and associated collateral cost under the current digital twin state. This evaluation provides the response-related quantities used by the decision model to assess the suitability of the candidate action before it is passed to the simulated response pathway. The procedure therefore provides a model-based evaluation of candidate actions rather than direct execution of a countermeasure.

The result of this evaluation is fed back to the decision layer before the next system state is generated. This procedure represents a model-based safety and consistency check within the simulation; it should not be interpreted as an operational safety certification, legal authorization, or substitute for human approval of active counter-UAS measures.

## 4. Proposed Methodology

The learning problem is determined by the sequence shown above. At each decision step, an edge agent will see its portion of the fused state, allocate its processing power, change its detection sensitivity and suggest a response option. These correlated decisions are formalized as a cooperative MAMDP and then the observation variables, actions, reward terms and MAPPO training procedure for the simulation are stated.

MAPPO was selected as the cooperative multi-agent controller because it supports centralized training with decentralized execution (CTDE), allowing each edge agent to execute decisions from its local observation while a centralized critic exploits the joint system state during training. This structure is compatible with the distributed edge architecture considered here and supports the mixed decision requirements of resource allocation, sensing adaptation, and response recommendation. The objective of this study is therefore not to propose a new MARL algorithm or establish MAPPO as superior to all alternative MARL methods, but to investigate how a cooperative MARL controller can be integrated with synchronized digital twin state information and edge-assisted counter-UAS decision-making.

### 4.1. Multi-Agent Markov Decision Process Formulation

Detection and response are modeled as a single Markov decision process (MDP) with multiple cooperating agents, as each edge node only sees a subset of the sensing and resource state.

In the present implementation, the four agents represent four edge processing nodes rather than the four sensing modalities. Radar, EO/IR, RF, and acoustic sensing are therefore not assigned to four separate MARL agents. Instead, the heterogeneous sensor observations are aligned and fused into the track-level digital twin state, as described further in the Section titled Multimodal Observation Alignment and Fusion, and each edge agent receives the portion of this synchronized state that is relevant to its assigned sensing and processing region. The four-agent configuration consequently models distributed edge decision-making over the protected area rather than modality-specific decision-making.

The local observation of agent $i,\;o_{i}(t)$, consists of its locally available track information, including target kinematic and confidence information derived from the fused sensing state, together with the agent’s local computational load and available response-resource state. Based on this observation, the actor produces action $a_{i}(t)$, which controls the decision variables assigned to that edge node, including sensing/processing-resource allocation and response recommendation. Agents do not exchange raw radar, EO/IR, RF, or acoustic streams. Coordination is based on processed track-state and resource-state information maintained through the synchronized digital twin. During centralized training, the critic has access to the joint state and joint actions of the agents, whereas during decentralized execution, each actor selects its action from its local observation.

(2) $$\begin{array}{c} M=\langle N,\;S,\{O_{i}{\}}_{i}\in N,\{A_{i}{\}}_{i}\in N,T,R,\gamma \rangle \end{array}$$

Here, N = {1, 2, …, n} identifies the edge agents. The global state S includes tracks, sensor-derived features, node-resource variables, and environmental terms. Each agent observes $O_{i}(s)$ and selects an action from $A_{i}$. The transition model T returns a distribution over next states under the joint action, and all agents receive the same reward R. The reported runs use a discount factor of γ=0.99.

Because the agents cooperate, they optimize one joint policy, $\pi^{*}=({\pi^{*}}_{1},{\pi^{*}}_{2},\;\ldots ,\;{\pi^{*}}_{N})$, rather than separate competing objectives:

(3) $$\begin{array}{c} \pi^{*}=\arg\max E\left[\sum_{t=0}^{T-1}\gamma^{t}R\left(s_{t},a_{1,t},\ldots ,a_{n,t}\right)\right] \end{array}$$

In Equation (3), $s_{t}$ represents the overall state at decision step t and $a_{1,t}$ represents agent i’s chosen action. The reward is for quick and correct detection and response; the penalty is for wasting resources and excessive false alarms [27].

### 4.2. Observation Space

At each decision step, agent i receives a local observation $O_{i}^{t}=O_{i}$($s_{t}$). The paper constructs this observation by concatenating four feature groups:

(4) $$\begin{array}{c} o_{i}^{t}=\left[f_{i}^{sensor},f_{i}^{track},f_{i}^{res},f_{i}^{coop}\right] \end{array}$$

The sensor feature vector is a fixed-length representation of the processed sensing information available to edge agent i within its assigned sensing and processing region. Depending on the sensing modalities contributing observations to that region, this vector may include encoded radar range-Doppler features, EO/IR visual features, RF spectral-power descriptors, and acoustic spatial-spectrum features. These modality-specific observations are processed and integrated into the synchronized track-level representation described in Section Multimodal Observation Alignment and Fusion; they do not correspond to separate modality-specific MARL agents. The resulting features are normalized before being supplied to the policy network. Each feature vector is run through the statistics to normalize it before feeding into the policy network.

The track component stores the track where the most K = 10 tracks in the sector that are observed by an agent. Three coordinates for position, three for velocity and one classification-confidence value are stored in each occupied slot. A zero padded slot has a binary mask to prevent padding from getting confused with a real track where the feature may be equal to zero.

The resource component includes four measurements of the resources at the local level: GPU, remaining GPU memory, current inference delay, and pending-frame queue. These values allow the actor to behave differently to the same uncertain track whether it has capacity to accumulate or not.

The cooperation part is purposefully small. The edge aggregator forwards the latest threat assessment and detection confidence to the other agents it has a relationship with. The actors do not pass entire local feature tensors at each decision point, thus avoiding an extra-high bandwidth workload.

### 4.3. Action Space Design

Every 0.1 s, an agent outputs one composite action consisting of a continuous compute allocation and two discrete operational choices: sensitivity and response.

(5) $$a_{i}^{t}={(a}_{i}^{res},\;a_{i}^{det},\;a_{i}^{resp})\;$$

The resource action is on a two-simplex, and separates the available GPU cycles into preprocessing, fusion/tracking and threat classification, with α1, α2 and α3 being required to sum to 1. This type allows the reallocation to be done over time when the track situation changes, instead of the controller having to select one specific compute mode.

The detection action has three sensitivity levels: low (0.8), medium (0.5), and high (0.3). Reducing the threshold will keep the low signal but will also make it easier to receive false alarms. The decision is assessed in conjunction with the evidence and the load of local processing.

The response action comprises alert and track, RF jamming, GPS spoofing, kinetic interception and a combination of all of these. An active action can only be taken once fused confidence is above a particular value, which has been chosen as 0.7. If this is not reached, the simulator takes over and continues to monitor the candidate.

### 4.4. Reward Function Design

All agents get the same reward as the system, which is considered successful if any node is successful, not necessarily all of them. Its terms require early recognition of a true intrusion, a good suggested response, cost-effectiveness in computing resources, and verification by multiple sensor channels.

(6) $$\begin{array}{c} R\left(s^{t},a^{t}\right)=w_{1}R^{det}+w_{2}R^{resp}+w_{3}R^{eff}+w_{4}R^{coop} \end{array}$$

For the experiments, the four reward weights are $w_{1}=1.0$, $w_{2}=0.8$, $w_{3}=0.1$, and $w_{4}=0.3$. The influence of the detection term is revisited in the ablation results in Section 6.

1. Detection Reward ($R_{det}$): The higher the reward that can be gained for an intrusion when it is detected earlier following geofence entry, the better.

(7) $$\begin{array}{c} R^{det}=\beta \frac{1}{{∆t}^{det}}\;\;\;\;\;\;\;\;\;(\mathrm{for}\;\mathrm{true}\;\mathrm{positives}) \end{array}$$

In Equation (7), ${∆t}^{det}$ denotes the time from geofence entry to detection and β=10 controls the scale. If Category I, II, or III is missing, the threat is penalized by −5, −10, or −20, respectively. A false alarm contributes a penalty of −2.

2. Response Effectiveness Reward ($R^{resp}$): The response term combines the digital twin’s estimated probability of response success with the modeled collateral-cost penalty:
  (8) $$\begin{array}{c} R^{resp}=\eta \cdot P\left(success|a^{resp},s\right)-\lambda \cdot C\left(collateral|a^{resp},s\right) \end{array}$$

Both terms are generated by the simulator response models using the current synchronized digital twin state, as described further in Section 3.4.3; they should therefore be interpreted as model-derived estimates rather than empirically measured physical-response outcomes.

The success term is a self-assessment by the twin of the effectiveness of the response to cancel or deflect the target. The collateral term is some sort of estimated disruption to the protected operations, such as interference or jamming. In the following evaluation, η=15 and λ=5 were chosen.

3. Resource Efficiency Penalty ($R_{eff}$): To discourage the controller from allocating excessive computational resources to every sensing stream, a resource-utilization penalty is defined as follows:
  (9) $$\begin{array}{c} R_{eff}=-\mu \frac{1}{n}\sum_{i=1}^{n}u_{i}^{t} \end{array}$$
  where $u_{i}^{t}$ denotes the normalized computational-resource utilization of agent i at time t, n is the number of agents, and μ=0.5 is the resource-utilization penalty coefficient. This term penalizes unnecessary computational-resource consumption while allowing additional resources to be allocated when they contribute to the primary detection and response objectives.

4. Cooperation Bonus ($R_{coop}$): If two modalities are able to detect the same event within two seconds, a +3 bonus is added for each confirmation. This is not about giving incentives for more and more confidence in the same stream, but for the confirmation.

The nominal weights reflect the operational priorities assumed in the present study rather than parameters obtained from a formal optimization procedure. Detection is assigned the highest priority because failure to identify a threat prevents subsequent response decisions, while response effectiveness is given the next-highest emphasis. Resource efficiency and inter-agent cooperation act as secondary objectives that discourage excessive resource consumption and uncoordinated decisions without dominating the primary detection and response objectives. These values should therefore be interpreted as design parameters for the modeled counter-UAS scenario rather than universally optimal reward coefficients. Their influence on policy performance is examined through the reward-weight sensitivity analysis reported in Section 6.5.2.

### 4.5. Cooperative Multi-Agent Proximal Policy Optimization

The cooperative controller is trained using MAPPO [27], which extends the PPO framework [33] to cooperative multi-agent settings under a CTDE structure [34]. The critic views the entire simulated state while training. By contrast, the deployed actor formulation only relies on local observations, and thus encourages distributed inference at the edge nodes, consistent with the desired distribution.

Decentralized Actor Networks: Agent i follows $\pi_{\theta_{i}}\left(a_{i}\;|o_{i}\right)$. It is based on fully connected layers with 256, 128 and 64 units, ReLU activations and layer normalization in its encoder. Two categorical heads and a Dirichlet head are used for allocating the simplex. This maintains a common feature representation, with each decision component being required to output its desired form.

Centralized Critic Network: Critic $V_{\emptyset }(s)$ (training time) receives everything from the local critics (observations) plus the active-track count, mean GPU, aggregate threat level, elapsed time since the last intrusion, etc. It has a 256-128-64 hidden-layer structure, similar to the actor, and outputs an estimate of a scalar number. In the case where trained actors are used, there is no need for the critic to be present during the inference.

Generalized Advantage Estimation (GAE): To minimize the variance of policy-gradient estimates, while keeping the bias manageable, this paper uses GAE(λ) [35]:

(10) $$\begin{array}{c} \delta_{t}=r_{t}+\gamma V_{\emptyset }\left(s_{t+1}\right)-V_{\emptyset }\left(s_{t}\right) \end{array}$$

(11) $$\begin{array}{c} A_{t}^{GAE}=\sum_{l=0}^{T-t-1}{\left(\gamma \lambda \right)}^{l}\delta_{t+l} \end{array}$$

where $\delta_{t}$ is the temporal-difference error, γ=0.99, and λ=0.95. The advantages are standardized within each training batch before an update is applied.

PPO Clipped Surrogate Objective: In each agent actor update, the standard clipped PPO objective is maximized:

(12) $$\begin{array}{c} r_{t}^{i}\left(\theta_{i}\right)=\frac{\pi_{\theta_{i}}\left(a_{t}^{i}\;|{\;o}_{t}^{i}\right)}{\pi \theta_{i}^{old}\left(a_{t}^{i}\;|{\;o}_{t}^{i}\right)} \end{array}$$

The ratio $r_{i}(\theta )$ compares the probability of the sampled action under the updated actor policy with that under the previous policy. The paper use a clipping parameter of ε=0.2 to limit the magnitude of each policy update and improve the training stability. The clipped surrogate objective for agent i is therefore defined as follows:

(13) $$\begin{array}{c} L_{i}^{CLIP\left(\theta_{i}\right)}=E_{t}\left[min\left(r_{t}^{i}\left(\theta_{i}\right)A_{t}^{GAE},clip\left(r_{t}^{i}\left(\theta_{i}\right),1-\varepsilon ,1+\varepsilon \right)A_{t}^{GAE}\right)\right] \end{array}$$

The centralized critic is trained by minimizing the value-estimation loss:

(14) $$\begin{array}{c} L^{V}\left(\emptyset \right)=E_{t}\left[{\left(V_{\emptyset }(S_{t})-R_{t}^{target}\right)}^{2}\right] \end{array}$$

To encourage exploration during training, an entropy term is included with coefficient $c_{2}=0.01$. The combined training objective for agent i is as follows:

(15) $$\begin{array}{c} L_{i}\left(\theta_{i},\emptyset \right)=L_{i}^{clip}\left(\theta_{i}\right)-c_{1}L^{V}\left(\emptyset \right)+c_{2}H\left({\pi \theta }_{i}\right) \end{array}$$

The learning rate of Adam to $3\times 10^{-4}$ is set with a batch size of 4096 transitions and 15 PPO epochs per update. The budget of the training is $5\times 10^{6}$ steps per environment. Each edge agent is represented by an actor policy $\pi_{\theta_{i}}$ operating on its local observation, while the centralized critic uses the joint system state during training. The actor networks use the same architecture but maintain agent-specific policy parameters in the formulation presented here [27]. All other main settings are γ=0.99, and λ=0.95. We show the EdgeTwin-DRL training procedure in Algorithm 1.
**Algorithm 1.** EdgeTwin-DRL Training Procedure$\mathrm{Input}:\;\mathrm{Digital}\;\mathrm{twin}\;\mathrm{environment}\;E_{DT}$, number of agents n$,\;\mathrm{training}\;\mathrm{steps}\;T_{max}$$\mathrm{Output}:\;\mathrm{Trained}\;\mathrm{actor}\;\mathrm{policies}\;\{{\pi \theta }_{1},\ldots ,{\pi \theta }_{n}\}$ 1.$\mathrm{Initialize}\;\mathrm{actor}\;\mathrm{network}\;\mathrm{parameters}\;\theta_{1},\ldots ,\theta_{n}$ randomly 2. Initialize centralized critic parameters ∅ randomly 3.$\;\mathrm{Initialize}\;\mathrm{digital}\;\mathrm{twin}\;\mathrm{airspace}\;\mathrm{model}\;E_{DT}$ 4.$\;\mathrm{for}\;\mathrm{episode}\;=\;1\;\mathrm{to}\;T_{max}$/$L_{ep}$ **do** 5.$\;\;\;\mathrm{scenario}\;\leftarrow \;\mathrm{SampleThreatProfile}(E_{DT}$) // DT generates scenario 6.$\;\;\;s_{0}\;\leftarrow \;E_{DT}.Reset\left(scenario\right)$ // Initialize airspace state 7.$\;\;\;\mathrm{for}\;t\;=\;0\;\mathrm{to}\;L_{ep}-1$ **do** 8.     for each agent i∈N **do** 9.       $O_{t}^{i}\leftarrow O_{i}(s_{t})$ // Extract local observation 10.      $a_{t}^{i}\sim \pi \theta_{i}\left(\cdot |O_{t}^{i}\right)$ // Sample action from policy **11.**     **end for** 12.    $a_{t}\leftarrow \left(a_{t}^{1},\ldots ,a_{t}^{n}\right)$ // Form joint action 13.    ${(s}_{t+1,}r_{t})\leftarrow E_{DT}.Step\left(s_{t},a_{t}\right)$ // DT simulates transition 14.$\;\;\;\;\mathrm{Store}\;(s_{t},{\left\{O_{t}^{i}\right\}}_{i\in N},{\left\{a_{t}^{i}\right\}}_{i\in N},r_{t},s_{t+1})$ in buffer *B* **15.**   **end for** 16.  // --- Policy Update Phase --- 17.$\;\;\mathrm{Compute}\;V\emptyset (s_{t})$ for all t using centralized critic 18.$\;\;\mathrm{Compute}\;\mathrm{GAE}\;\mathrm{advantages}\;A_{t}^{GAE}$ using Equations (10) and (11) 19.$\;\;\mathrm{Normalize}\;\mathrm{advantages}:\;A_{t}^{GAE}$ ← ($A_{t}^{GAE}$ – mean($A^{GAE}$))/std($A^{GAE}$) 20.   **for** epoch = 1 to 15 **do** 21.     **for** each mini-batch in B **do** 22.      for each agent i∈N **do** 23.$\;\;\;\;\;\;\;\;\mathrm{Compute}\;r_{t}^{i}(\theta_{i})$ using Equation (12) 24.$\;\;\;\;\;\;\;\;\mathrm{Compute}\;L_{i}^{CLIP}(\theta_{i})$ using Equation (13) **25.**        **end for** 26.$\;\;\;\;\;\;\mathrm{Compute}\;L^{V}(\emptyset )$ using Equation (14) 27.$\;\;\;\;\;\;\mathrm{Update}\;\theta_{i}\leftarrow \theta_{i}+\alpha {∆}_{\theta_{i}}\left[L_{i}^{CLIP}(\theta_{i})+c_{2}H\right](\pi_{\theta_{i}})$ 28.$\;\;\;\;\;\;\mathrm{Update}\;\emptyset \leftarrow \emptyset -\alpha_{c1}\nabla_{\emptyset }L^{v}(\emptyset )$ **29.**     **end for** **30.**   **end for** 31.  Clear buffer B **32.** **end for** **33.** $\mathrm{return}\;\{\pi_{\theta 1},\ldots ,\pi_{\theta n}\}$

### 4.6. Digital Twin Integration in Training and Deployment

There are two places in the workflow where the twin is present: when policies are being learned and when generating trajectories and rewards. When simulating operation, it lets you know the state of the system and carries out a brief model-based check before recommending an action.

Training Phase—Environment Simulation: One to five drones, one of four path families (straight approach, loitering, evasive zigzag, or coordinated multi-vector motion), a speed of 10 to 80 km/h, an RF mode and weather and visibility values are selected for the episode. The radar range equation, the Swerling-I fluctuation, and the log-normal clutter are used to generate radar observations, while the atmospheric transmission is used for the EO/IR observations, the path loss and log-distance fading are used for the RF observations and the spreading and the frequency-dependent attenuation are used for the acoustic observations [14,16]. These decisions are not based on measurements taken by deployed sensors but rather on plausible training inputs for the simulator.

When an episode is initialized in such a way that learning does not rely on a single set of model parameters, it is known as domain randomization [36]. The noise level is disturbed by ±20%, the detection range by ±15%, the clutter density by ±30%, and the radar cross section by ±25%. The training starts with single-drone scenarios in good weather conditions, and then gradually incorporates adverse weather, evasive routes, and coordinated training.

In deployment-phase operational decision support, actors receive observations from a twin deployed on the edge tier. For an active response, it is proposed to conduct a short forward-evaluation (here 50–100 ms) and to compare the estimation of success with a threshold (here 0.6). There may be a time when quiet-period site statistics can help to control fine-tuning [10] but this is not shown in the present study, nor is the physical response path.

### 4.7. Computational and Communication Complexity

The computational requirements of EdgeTwin-DRL arise primarily from multimodal observation processing, digital twin state updating, and the actor–network inference. Let N denote the number of active UAV tracks, M the number of sensing modalities, A the number of edge agents, and $P_{actor}$ the number of parameters in an actor network. Track-level multimodal processing requires observations from up to M modalities to be associated with the N active tracks, while the synchronized digital twin maintains the resulting track and resource states. Consequently, the online state-processing workload increases with the number of active tracks and available sensor observations. Actor inference scales with the size of the actor network and is performed independently by the participating edge agents.

The centralized critic is required during MAPPO training but not for decentralized actor execution. Thus, the larger joint-state processing associated with centralized training does not need to be executed by every edge agent during deployment. Nevertheless, increasing the number of agents enlarges the joint representation processed during training and increases the coordination overhead, which is consistent with the degradation observed for the eight-agent configuration in the scalability analysis.

Communication overhead is limited to exchanging processed observations, track-state updates, resource status, and coordination information rather than continuously transmitting raw radar, EO/IR, RF, or acoustic streams among all agents. If each agent communicates a state message of size $B_{s}$ at an effective update rate $f_{s}$, the aggregate inter-agent/twin communication requirement can be approximately represented as

(16) $$\begin{array}{c} C_{common}\propto {AB}_{s}f_{s} \end{array}$$

excluding protocol-specific headers and retransmissions. Memory requirements are primarily determined by the actor parameters, maintained digital twin track states, observation buffers, and processing queues. During centralized training, additional memory is required for the critic and rollout buffers. Because the present experiments emulate the edge nodes on a workstation rather than physical Jetson devices, hardware-specific memory consumption and per-module execution times are not reported as measured deployment quantities. These aspects require profiling on the target edge hardware during the hardware-in-the-loop validation stage.

## 5. Experimental Setup

All quantitative performance results reported in our study are discussed in this section. The publicly available RF datasets are used for calibration and independent validation of the RF sensing models, while the remaining sensing components are parameterized using published experimental measurements. These data sources improve the empirical grounding of the simulated observation models but do not constitute an end-to-end real-world validation of EdgeTwin-DRL. Accordingly, the reported detection, response, latency, and resource-utilization results should be interpreted as simulation-based evaluations of the proposed architecture.

### 5.1. Computing Platform

Experiments were conducted on a workstation equipped with an Intel Core i9-13900K processor (24 cores, up to 5.8 GHz) (Intel, Santa Clara, CA, USA), 64 GB of DDR5 memory, and an NVIDIA RTX 4090 GPU (Nvidia, Santa Clara, CA, USA) with 24 GB of GDDR6X memory. The simulator and learning framework were implemented using Python 3.10, PyTorch 2.1, and Stable-Baselines3 2.1.0. The four edge agents were emulated as separate processes sharing the workstation GPU, while per-node computational capacity, processing queues, and resource constraints were imposed within the simulator rather than by the physical workstation hardware. Inter-node communication delay was sampled from a log-normal distribution with a mean of 0.3 ms and a standard deviation of 0.1 ms based on representative 10 Gbps Ethernet measurements [37]. For the Cloud-DRL baseline, round-trip communication delay was uniformly sampled between 50 and 200 ms.

The simulated end-to-end detection-to-response latency comprises sensor/observation processing, inter-node or cloud communication, policy inference, digital twin state updating and candidate-response evaluation. For EdgeTwin-DRL, communication occurs within the modeled edge environment, whereas Cloud-DRL additionally incurs the sampled 50–200 ms cloud round-trip delay. Consequently, the latency advantage of EdgeTwin-DRL over Cloud-DRL depends directly on this communication-delay assumption and should not be interpreted as a hardware-measured or universally fixed reduction.

The NVIDIA Jetson Orin platform [32] was considered as the target edge-device class for parameterizing the computational resource constraints represented in the simulator; however, no physical Jetson device was used in the reported experiments. Accordingly, the edge-processing constraints, resource utilization, and associated timing behavior represent simulation-derived estimates under the modeled edge-computing assumptions rather than measurements obtained from deployed Jetson hardware.

Actor network inference was executed on the RTX 4090 workstation as part of the simulation. Therefore, the end-to-end latency values reported in Section 6 combine simulator-generated processing and communication components and should be interpreted as simulation-based latency estimates, not as experimentally measured latency on a Jetson Orin or other deployed edge platform. Physical edge-device benchmarking is reserved for the hardware-in-the-loop validation stage discussed in Section 7.

### 5.2. Real-World Datasets for Sensor Model Calibration

Calibration is essential for ensuring that the simulated sensor observations closely resemble those obtained from real UAV sensing systems. In this study, two publicly available radio-frequency (RF) datasets were employed exclusively for the calibration and independent validation of the RF sensing component within the proposed simulation environment. No machine learning models were trained directly using these datasets; instead, they were used to calibrate the RF observation models and verify that the simulated RF characteristics were consistent with measurements collected from real UAV communications.

The first dataset is the DroneRF Dataset [38], which contains RF recordings collected from several commercially available UAV platforms operating under multiple flight modes in the 2.4 GHz ISM band. The dataset was used to calibrate the RF sensing model by matching the distributions of received signal power, spectral bandwidth, modulation characteristics, and inter-packet timing observed in the simulated environment with those reported in the real measurements. Following calibration, the simulated RF observations achieved a mean Kullback–Leibler (KL) divergence below 0.05 across the evaluated operating modes, indicating close agreement between the simulated and measured RF signal distributions.

To further evaluate the generalization capability of the calibrated RF sensing model, the publicly available DroneRFa dataset [39] was employed, which provides a large-scale collection of RF signals captured from low-altitude UAV communications under diverse operating conditions. Unlike the DroneRF dataset, which was used for parameter calibration, DroneRFa served exclusively as an independent validation dataset. The comparison focused on RF fingerprint characteristics, spectral distributions, and received signal statistics to verify that the simulated RF observations remained consistent with measurements obtained from the UAV communications not used during the calibration process. This cross-dataset validation reduces the risk of overfitting the simulation model to a single RF dataset and provides a more objective assessment of its generalization capability.

Only the RF sensing component utilizes publicly available datasets for calibration and independent validation. The radar, EO/IR, and acoustic sensing models were calibrated using published experimental measurements and sensor performance characteristics reported in the literature rather than publicly available datasets.

For the remaining sensing modalities, the radar model was calibrated using the FMCW detection measurements reported by Han et al. [14], resulting in a detection-probability residual below 0.04 across signal-to-clutter intervals between 0 and 20 dB, while the simulated false-alarm rate remained within ±0.5% of the reported operating point. The acoustic sensing model was calibrated using the effective detection range and bearing-error measurements reported by Ding et al. [16], producing an average range error of approximately 8% and a bearing error of about 1.5°. The EO/IR sensing parameters were derived from published atmospheric-transmission models and commercial electro-optical detection-range specifications. The calibrated parameters and corresponding residual analyses will be included in the released implementation to facilitate reproducibility.

### 5.3. Digital Twin Simulation Environment

The twin is a custom multi-agent environment, which is compatible with Gymnasium [38]. It is a subject area of 5 km × 5 km around a specified facility, and 0.5 km high. A 300 s episode will have 3000 policy decisions, as it is stepped at 10 Hz. With the RTX 4090 GPU from this study, vectorized simulation can provide up to 64 episodes running concurrently, and achieve roughly 45,000 environment steps per second.

The simulator has one observation model for each of the sensors in Section 3.2. The radar range equation and Swerling-I fluctuation, log-normal clutter and additive white Gaussian noise are used as radar samples, and the radar cross-section is sampled from the range 0.001–0.1 m^2^, which is reported in counter-UAS work [5]. EO/IR samples are based on a simplified atmospheric-transmission term, thermal contrast and apparent target size. Path loss, intermittent transmission and log-distance shadow fading with 2.2–3.5 environment dependent exponent are examples of RF samples. The acoustic samples make use of the spherical spreading, the frequency-dependent absorption according to ISO 9613-1:1993 and an environment-specific background-noise profile.

Episodes are built out of the three categories of threats that are created in Section 3.1. For category I, one or two slow surveillance tracks with active RF transmissions are produced. Category II will produce from one to three direct approaches with speeds ranging from 40 to 80 km/h, and 30% of the cases generated by Category II will have RF silence. Category III creates three to five coordinated tracks coming from various angles and is conducting evasive maneuvers. The curriculum includes the use of Category I in the first 1 million steps, followed by Category II for steps 1–3 million, and sampling all three categories uniformly in the final 2 million steps. Also randomized between episodes are the weather types and visibility, numerical values of which are given in Table 2, and the time of day.

### 5.4. Baseline Methods

The contribution of edge placement, multi-agent coordination and the calibrated twin are separated using five comparisons. Both methods are tested in the same simulator, and their scenario generation is the same.

#### 5.4.1. Cloud-Centralized DRL (Cloud-DRL)

A centralized server receives the sensor data from a single PPO agent [33] and decides on the detection, allocation and responses. The delay for communication is set to 50~200 ms and Compute is assumed to not be constrained. This comparison only considers the latency associated with using a remote decision point that is modeled.

#### 5.4.2. Independent PPO (IPPO)

All the edge nodes run PPO independently [27]. Agents receive the reward signal but do not use a centralized critic or use global state. IPPO thus quantifies the added value of overt coordination in the MAPPO design.

#### 5.4.3. Multi-Agent Deep Deterministic Policy Gradient (MADDPG)

This is the off-policy multi-agent actor–critic baseline based on Lowe et al. [34], which takes all agents’ observations and actions as its conditioning, and has a replay buffer of 10^6^ transitions. It offers an off-policy comparison of the cooperative task.

The last one is a baseline known as Rule-Based Expert System (RB-Expert), where the detection sensitivities are static, the mapping from threat category to response is deterministic, and resources are equally distributed among the edge nodes. To get a competitive non-learning comparison, thresholds and mappings are chosen by simulating a grid search [40].

#### 5.4.4. Rule-Based Expert System (RB-Expert)

This non-learning baseline uses fixed detection sensitivities, deterministic mappings from threat categories to response recommendations, and equal resource allocation among the edge nodes. Thresholds and response mappings are selected through a grid search in the simulator [40] to provide a competitive rule-based comparison.

#### 5.4.5. MAPPO Without Digital Twin (MAPPO-NoCalib)

This ablation retains the MAPPO controller, synchronized state representation, and simulated operational environment used by EdgeTwin-DRL, but replaces the calibrated and environment-dependent sensor observation models with fixed sensor-noise and detection models. It therefore isolates the contribution of calibrated and environment-dependent sensing rather than removing the complete digital twin environment.

The MARL baselines are intended to evaluate the proposed architecture against representative independent and centralized-training/decentralized-execution controllers rather than to establish MAPPO as the universally superior MARL algorithm. HAPPO, QMIX, QPLEX, and MATD3 were not included in the present experimental scope because they introduce different policy-factorization, action-space, or optimization assumptions and would require separate implementation and tuning for a controlled comparison. Accordingly, conclusions from the baseline comparison are restricted to the methods evaluated in this study.

### 5.5. Evaluation Metrics

A faster system is not evaluated if the report metrics related to the detection quality or response outcome have not been reported, and that is where the six metrics are shown.

Detection Accuracy (DA): DA is the ratio of the number of airspace frames classified correctly. In addition, the F1-score is reported to provide a summary of the precision and recall balance when there is class imbalance, as normal non-intrusion frames are more than the intrusion frames.

False-Positive Rate (FPR): The FPR is calculated for the frames where an intrusion is not present as FP/(FP + TN). This value is significant when operating, as useless alerts need to be acted upon and could start an unnecessary review of the response. False detections are also penalized during policy training through the false-alarm term in the detection reward. Thus, increasing sensitivity is not rewarded independently of its effect on false positives, and FPR is reported alongside detection accuracy and F1-score to capture this trade-off.

Detection to Response Latency (Lat): This is the simulated time between detecting a geofence and sending a response command. It comprises the sensing, inter-node communication in the emulated scenario, pre-processing, fusion, actor inference, twin validation and command generation.

Response Success Rate (RSR): The outcome model considers the response successful if it predicts the intended response, e.g., retreat, forced landing or loss of control. RSR refers to decisions made in the simulator and should not be considered as a physical RSR.

Average GPU (GPU): During evaluation, the average utilization of edge nodes emulated on the GPU is measured. Lower utilization gives time for bursts of additional processing and/or hardware constraints.

Cumulative Reward ($R_{cum}$): This is the discounted return of an episode as given by Equation (6). It is used in conjunction with latency, detection measures and response outcome, and is a reflection of the goals chosen in the reward function.

### 5.6. Training Configuration and Hyperparameters

The same observation/act definitions are used for each DRL method, which are trained for $5\times 10^{6}$ environment steps. In multi-agent configurations, n=4 agents are used and the settings for MADDPG are taken from Lowe et al. [34]. The grid search method in the simulator is used to tune the fixed rule comparison. Each experiment is repeated using random seeds 0, 42, 123, 456, and 789 and the mean and standard deviation are reported. The other settings are shown in Table 3.

Each method was evaluated using five independent random seeds, with 100 evaluation episodes per seed, resulting in a total of 500 evaluation episodes per method. The same evaluation protocol was applied to all compared methods. Performance metrics were first computed over the evaluation episodes associated with each seed, and the reported mean and standard deviation were then calculated across the five seed-level results. Thus, the reported standard deviations characterize variation across independent random seeds rather than variation across individual episodes.

A reward-weight sensitivity experiment was additionally conducted to determine whether the performance of EdgeTwin-DRL depends strongly on the nominal weighting configuration. The nominal configuration was compared with alternative configurations that modify the relative emphasis placed on detection, response effectiveness, resource efficiency, and cooperation while leaving the remaining training and evaluation settings unchanged. Each configuration was trained and evaluated using the same protocol as the main EdgeTwin-DRL model. This analysis is intended to assess the robustness of the selected reward design rather than to identify a universally optimal set of coefficients.

## 6. Results and Discussion

First, a comparison is conducted for the methods across all simulated settings and then convergence, the effects of environment and threat type, reward-weight sensitivity, and agent scalability are examined. Unless otherwise noted, reported values are presented as the mean and standard deviation across five seeds and 500 test episodes per method. A reported value is the mean and SD from five seeds and 500 test episodes per method, unless otherwise noted.

### 6.1. Overall Comparative Performance

The three modeled environments are paired with the three threat categories in Table 4. In this protocol, the number value of all reported metrics is highest in EdgeTwin-DRL. The Wilcoxon signed rank test was used at the seed level to compare each of the specific comparison methods and the results were found to still be significant following Bonferroni correction for the five methods used.

For the combined test set, EdgeTwin-DRL produces a detection accuracy of 97.3% and an F1-score of 95.8%. The values reported in MAPPO-NoCalib are lower by 1.5 and 2.7 percentage points. In addition, EdgeTwin-DRL also trades off a false-positive rate of 1.4%, whereas MAPPO-NoCalib trades off 2.1% and RB-Expert trades off 6.8%. The latency values offer the greatest distance in this simulator: 527 ms for EdgeTwin-DRL, 713 ms for MAPPO-NoCalib, and 1910 ms for Cloud-DRL. The first two values are 26% and 72% reductions, respectively, from the two comparison methods. This reduction should be interpreted under the communication assumptions of the simulator. In particular, the Cloud-DRL baseline includes a uniformly sampled 50–200 ms round-trip communication delay, whereas EdgeTwin-DRL uses the modeled local inter-node communication described in Section 5.1. The observed 72% reduction therefore reflects both the computational architecture and the assumed difference between edge-local and cloud communication delays; different network conditions would change the magnitude of this advantage.

The Cloud-DRL comparison can still be useful in terms of detection quality but has the longest simulated response delay as it incorporates the communication round trip to a remote server. This outcome is only a consequence of that architecture in our simulator and does not mean that a cloud-based classifier would not be able to recognize drone observations.

### 6.2. Training Convergence Analysis

**Figure 2:**
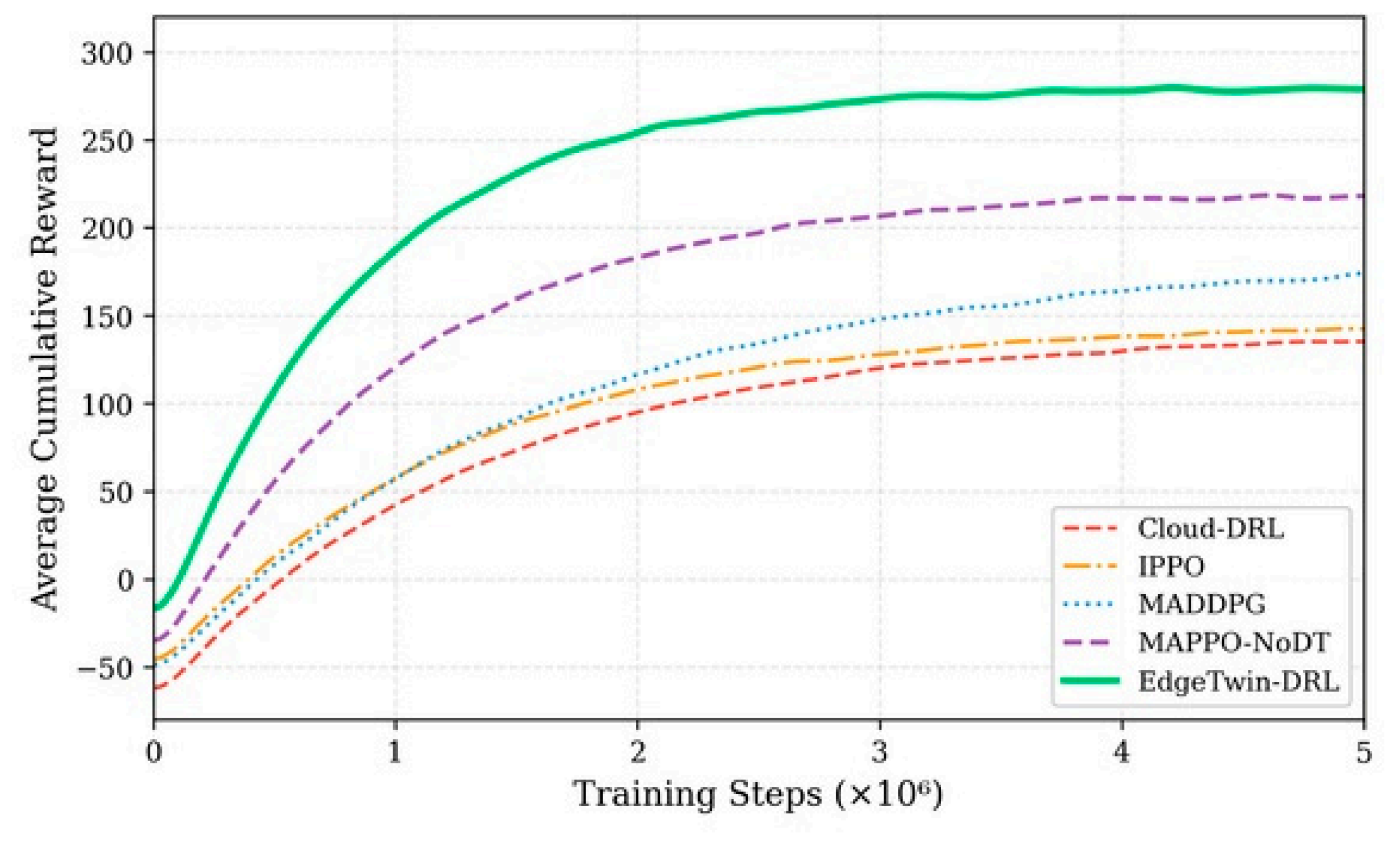
Training convergence comparison. EdgeTwin-DRL (green line) converges fastest to the highest cumulative reward. Cloud-DRL (red dashed line) is hampered by stochastic communication latency. MADDPG (blue dotted line) converges slower despite off-policy replay.

Figure 2 shows the total reward over 5 million steps of the environment. By about 2.8 million steps, EdgeTwin-DRL achieves 90% of its final reported reward and levels off at around 283. However, MAPPO-NoCalib does not increase as steeply and becomes flat at around 221. The difference is consistent with what is observed during training, which involves detecting environment-dependent sensor variation, and not fixed-noise detections.

In this model, stochastic communication delay is added to the transition sequence, and the convergence of Cloud-DRL is slower and obtains a lower reward. IPPO is better than Cloud-DRL in terms of learning but it is not as good as the MAPPO variants which are coordinated, indicating that there is some benefit to having a shared critic to distribute tasks. For these runs, MADDPG trains slower than the MAPPO variants, as seen in cooperative-task experiments by Yu et al. [27].

### 6.3. Scenario-Specific Performance

**Figure 3:**
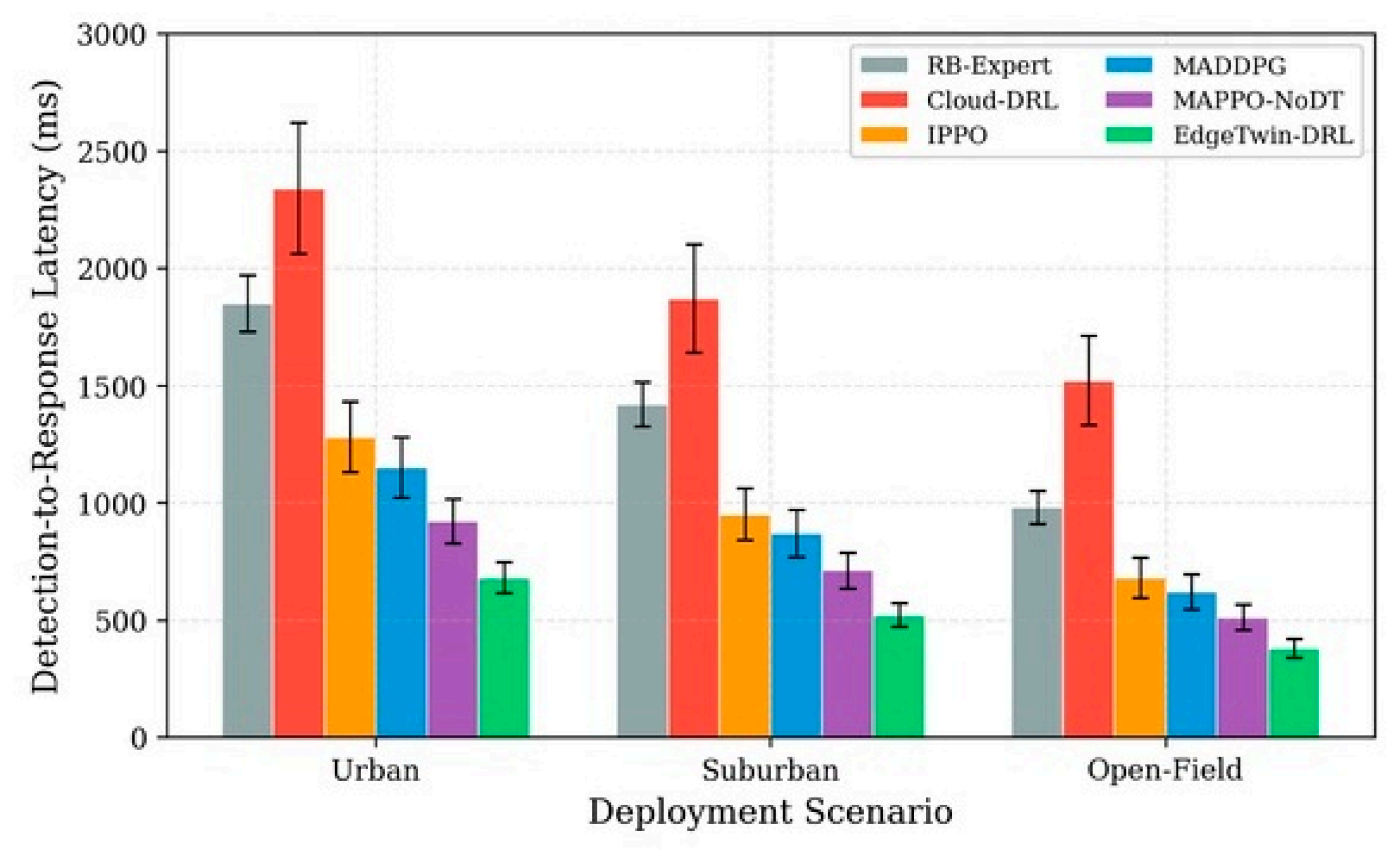
End-to-end detection-to-response latency across urban, suburban, and open-field deployment scenarios. Error bars indicate standard deviation over five seeds. EdgeTwin-DRL (green) achieves the lowest latency in all scenarios.

The response latency in the three simulated environments is compared in Figure 3. All methods slow down in the urban environment since the model adds clutter, noise and multipaths. In all three scenarios, EdgeTwin-DRL is still the lowest-latency method, with average latency times of 680 ms (urban), 520 ms (suburban), and 380 ms (open-field).

The greatest separation is in the urban setting. In this model, the ambiguity of radar evidence results in more processing effort for the RF and acoustic features, as revealed by the learned-policy traces. This pattern was discovered through observations made while simulating the operation of the system, not as an operating rule of an urban system. Physical data would be required prior to using the same behavior as a deployment recommendation.

### 6.4. Per-Threat-Category Analysis

**Figure 4:**
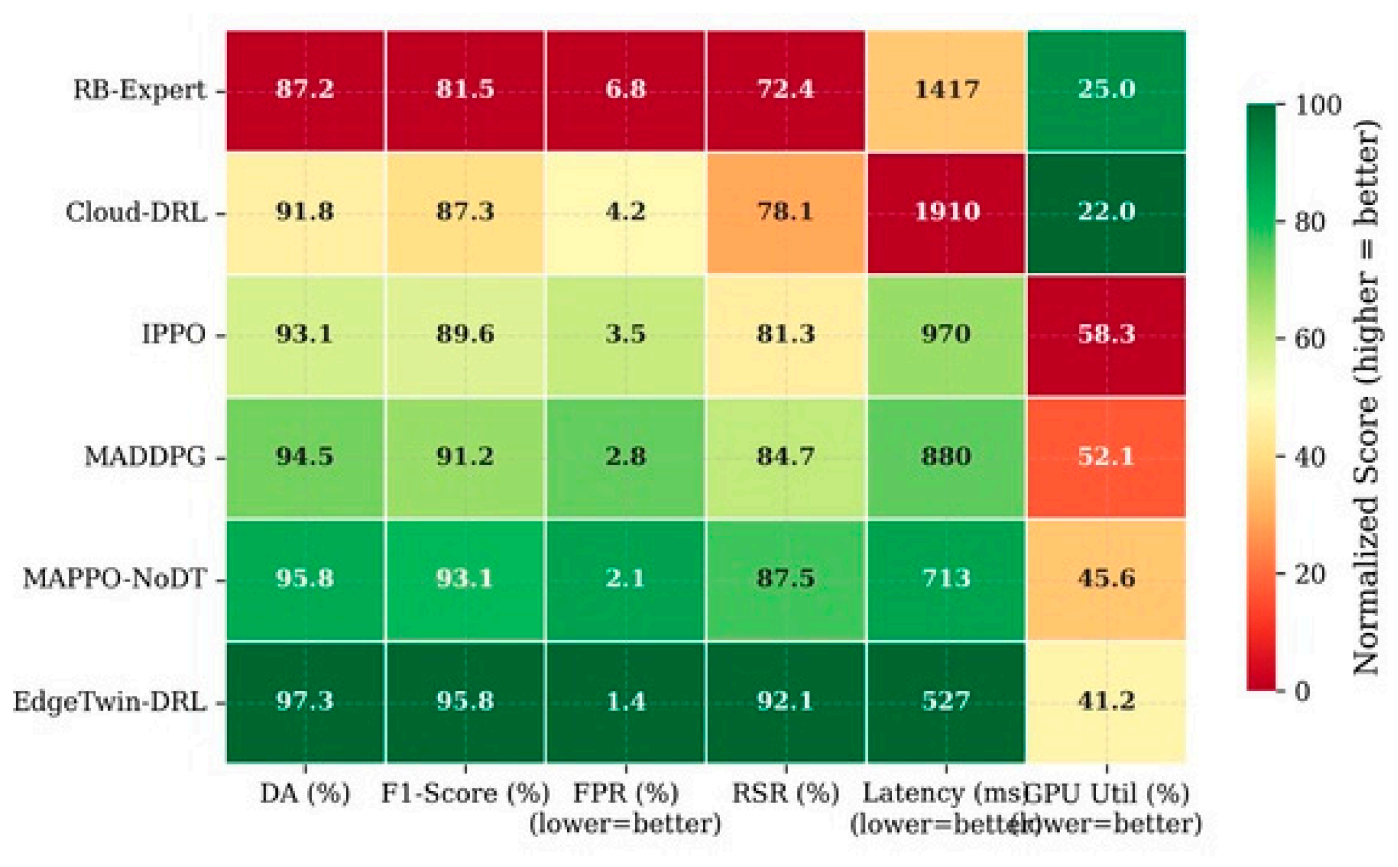
Normalized metric summary across the simulated settings. Numerical annotations show the compared values; the color scale is a visual aid.

The metrics are summarized after normalizing and averaging over the simulated settings in Figure 4. The shaded values are added for visual comparison only; the interpretation is based on the printed values.

**Figure 5:**
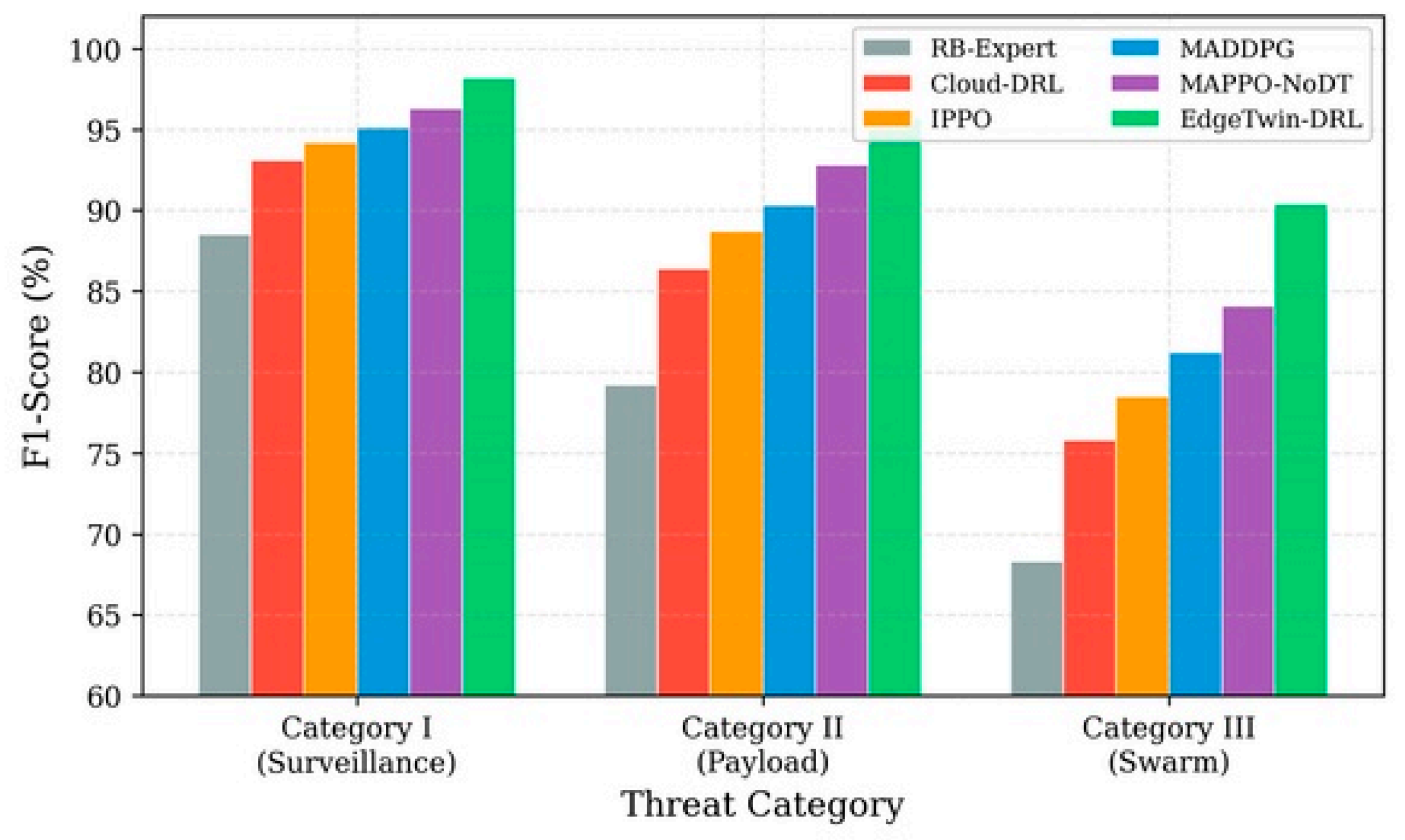
Simulated F1-score by threat category. The largest gap occurs in Category III: 90.4% for EdgeTwin-DRL and 68.3% for RB-Expert.

Figure 5 is a breakdown of F1-score by threat category. The biggest difference is found for the category III, coordinated tracks: EdgeTwin-DRL shows 90.4%, MAPPO-NoCalib shows 84.1% and RB-Expert shows 68.3% in the simulated evaluation.

Several tracks are active at the same time in Category III and there is a concurrent demand for processing and also the prioritization of response. With CTDE, they can be trained for complementary behaviors, and during the recorded runs, some actions promote continuity on the track while others put more focus on classification. Unlike IPPO, this training-time coordination is not possible with IPPO because there is no centralized critic. The findings imply that, for the family of simulated scenarios in this study, coordination is most helpful when multiple tracks are to be managed at once.

### 6.5. Ablation Studies

**Figure 6:**
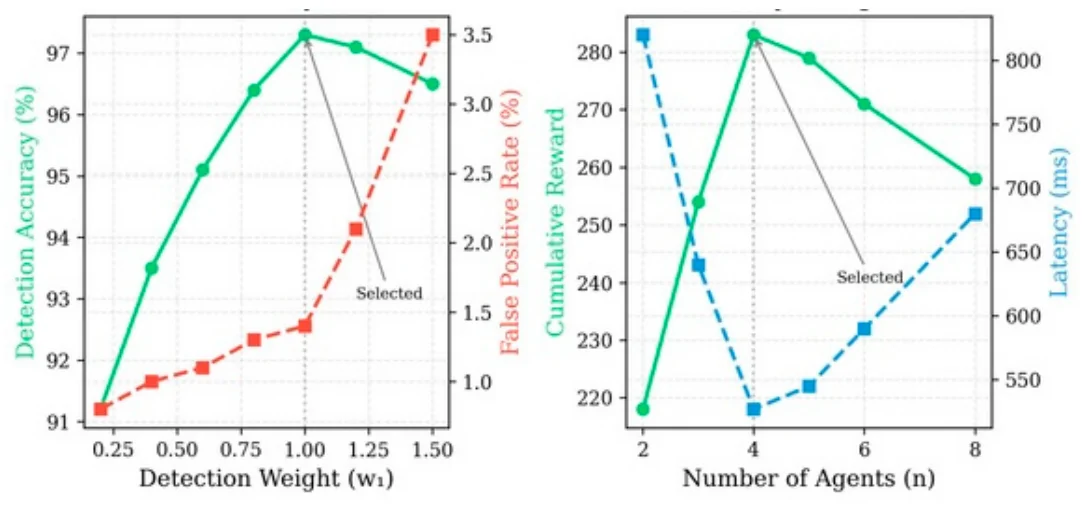
Sensitivity and scalability analysis of EdgeTwin-DRL. (**Left**) Effect of the detection reward weight ($w_{1}$) on detection accuracy (green solid line) and false-positive rate (red dashed line). (**Right**) Effect of the number of agents (n) on cumulative reward (green solid line) and latency (blue dashed line). The gray annotations indicate the parameter values selected for the reported experiments.

The ablation analysis examines two aspects of the proposed configuration: sensitivity to the detection-reward weight and scalability with respect to the number of edge agents. The corresponding results are summarized in Figure 6.

#### 6.5.1. Reward Weight Sensitivity (Figure 6, Left)

Detection accuracy rises from 91.2% at w_1_ = 0.2 to 97.3% at w_1_ = 1.0, before decreasing slightly to 96.5% at w_1_ = 1.5. FPR is between 0.8% and 1.4% up to w_1_ = 1.0, then increases to 3.5%. For these experiments, it has been reported that w_1_ = 1.0, a value that would denote the most aggressive detector to date, but at which the balance between warping and end-to-end quality would be best observed in these scenarios.

To evaluate whether the reported performance is dependent on the manually selected reward coefficients, the nominal reward configuration was compared with alternative configurations that independently increased or decreased the emphasis assigned to the principal reward components. The purpose of this analysis is not to exhaustively optimize the reward weights, but to determine whether moderate changes in their relative importance substantially alter the learned policy.

#### 6.5.2. Agent Scalability (Figure 6, Right)

Reward improves to 283 and average latency decreases to 527 ms when the number of agents is increased from two to four; the reward is decreased to 258 and the latency is increased to 680 ms if the number of agents is increased from four to eight. A possible explanation is that it involves greater representation of the joint in the centralized critic. In this study, four agents were employed; note that this is not an optimal setting for a larger perimeter nor another distribution of scenarios. It is vital to note that this trend is consistent with the complexity discussion in Section 4.7: increasing the number of agents enlarges the coordination and centralized-training workload, so additional agents do not necessarily translate into improved system-level performance.

### 6.6. Discussion

The results presented in this study should be interpreted within the scope of the calibrated simulation environment rather than as a field-validated counter-UAS performance. Three main observations can be drawn from the comparative evaluation. First, placing the immediate decision loop at the edge reduces the communication-delay component represented in the Cloud-DRL baseline. Second, cooperative multi-agent decision-making provides better performance than independently trained actors in the multi-track scenarios considered. Third, the configuration incorporating calibrated and environment-dependent sensing outperforms the corresponding fixed-noise ablation. These findings establish comparative differences among the evaluated architectures under the stated simulation models and assumptions, but they do not establish equivalent performance in a physical deployment.

Within the simulated decision process, the digital twin contributes to both state maintenance and decision evaluation. It maintains a synchronized representation of the modeled airspace and edge-resource state, provides the environment used for policy training, and evaluates candidate responses before they are passed to the simulated response pathway. As detailed in Section 3.4.3, the response-success probability and collateral-cost terms are model-derived quantities computed from the candidate response and the current synchronized state. These quantities are used for simulated action evaluation and should not be interpreted as measurements of the physical countermeasures’ effectiveness.

The comparison with the fixed-noise ablation indicates that incorporating calibrated and environment-dependent sensing positively contributes to the overall framework performance. However, the magnitude of this contribution under operational conditions cannot be established without hardware-in-the-loop and physical-system validation. Accordingly, the reported response-success rate and other performance measures should be regarded as simulator-derived estimates rather than measured countermeasure effectiveness.

The latency results require similar interpretation. The reduction relative to Cloud-DRL is influenced by the communication model adopted in the simulator, particularly the remote round-trip delay assigned to the cloud-centric architecture. Consequently, the reported latency reduction should not be interpreted as a universal edge-versus-cloud performance advantage. Physical deployment would additionally introduce hardware-specific inference time, communication jitter, sensor-interface delays, operating-system scheduling, and synchronization overhead that are only approximated in the present simulation.

The resource-utilization results also represent the computational assumptions of the modeled architecture. RB-Expert requires less GPU capacity because its computational demand is less dependent on adaptive learning workloads, but this lower resource demand is accompanied by a reduced detection and response performance within the simulator. An evaluation of the same trade-off on physical edge hardware would additionally require consideration of power consumption, thermal constraints, memory utilization, hardware scheduling, and sustained inference performance.

More generally, some degradation should be expected when transferring EdgeTwin-DRL from a simulation to a physical counter-UAS environment. Potential sources include sensor-calibration drift, unmodeled clutter and interference, environmental variability, imperfect temporal synchronization, communication jitter, hardware-specific processing delays, and threat behaviors outside the simulated distributions. The domain-randomization strategy used during training is intended to reduce dependence on a single set of environmental and sensing parameters and thereby improve robustness to deployment variability; however, it cannot eliminate the simulation-to-real gap [41]. Such degradation can be mitigated through hardware-in-the-loop evaluation, site-specific sensor calibration, controlled passive field trials, confidence and state-consistency monitoring, and progressive policy adaptation using observations representative of the deployment environment. These steps would allow model mismatch to be identified before active operation and provide a controlled transition from a simulation-based evaluation toward physical deployment.

The sensor-noise models should also be interpreted within the scope of the present system-level evaluation. The adopted radar clutter and additive noise formulations provide controlled and reproducible sensing conditions for comparing the evaluated architectures, rather than a complete statistical representation of physical sensor interference. Real RF, acoustic, and radar measurements may exhibit non-stationary, multiplicative, impulsive, or heavy-tailed characteristics. Such modality-specific noise modeling can be incorporated into the digital twin observation models without changing the proposed EdgeTwin-DRL architecture, but its physical characterization and validation are beyond the scope of our study.

The threat-model scope also imposes a limitation on the present evaluation. The simulated scenarios consider straight approaches, loitering behavior, evasive zigzag motion, and coordinated multi-vector incursions, but they do not explicitly represent highly unpredictable trajectories generated through chaotic dynamics. Such trajectories may exhibit strong sensitivity to initial conditions and non-repeating nonlinear motion, potentially placing greater demands on track prediction, multimodal sensor coordination, and response planning than the trajectory families evaluated here. Therefore, the reported robustness results should be interpreted as applying to the modeled threat families and their randomized variations rather than to arbitrary or previously unseen evasive UAV behaviors. Chaos-based and adaptive nonlinear trajectory generation are identified as important extensions for future robustness evaluation.

Large-scale coordinated UAV swarms represent a further extension beyond the threat scenarios evaluated in this study. The current architecture could be adapted to such scenarios by partitioning the protected airspace among multiple edge-agent clusters, maintaining local track subsets within the digital twin, and coordinating resource allocation and response recommendations across neighboring clusters. Attention-based or hierarchical multi-agent coordination could further restrict each agent’s decision context to locally relevant tracks while enabling higher-level coordination across the protected area. However, the present four-agent evaluation does not establish scalability to massive swarm attacks; such scenarios would require dedicated evaluation of track density, communication overhead, computational load, and coordination performance.

Finally, the observed improvements should be attributed to the system-level formulation and interaction among synchronized multimodal sensing, edge-resource allocation, cooperative decision-making, and model-based response evaluation, rather than to a new reinforcement-learning optimization algorithm. EdgeTwin-DRL uses MAPPO as an established learning method; the contribution of the present work lies primarily in its integration within an edge-assisted digital twin architecture for counter-UAS decision support.

#### Safety and Deployment Considerations

False-positive detections are particularly important in counter-UAS applications because an incorrect threat classification could lead to an unnecessary response recommendation. For this reason, the actions produced by our framework should be interpreted as decision support recommendations rather than autonomous authorization of active countermeasures. In our framework, low-confidence cases can remain under passive tracking, while candidate active responses are evaluated within the digital twin before being passed to the simulated response pathway. In a physical deployment, active measures such as RF jamming or navigation disruption would require human authorization and compliance with the applicable regulatory and safety requirements.

Further, digital twin inconsistency represents an additional deployment risk. Delayed observations, sensor faults, synchronization errors, or model mismatch may cause the maintained twin state to diverge from the physical environment. Operational deployment should therefore incorporate state-freshness and confidence monitoring so that active-response recommendations can be withheld when the synchronized state is stale, inconsistent, or insufficiently reliable. Such safeguards are not evaluated as a physical fail-safe mechanism in the present simulation and require validation with live sensing and edge hardware.

Lastly, the present simulator can represent reduced sensor observability by varying parameters such as radar cross-section, sensor SNR, detection range, clutter, RF availability, and environmental noise. However, the reported experiments do not explicitly model or validate against purpose-built radar-signature- or acoustic-signature-reduction technologies. Such techniques could be incorporated in future evaluations by modifying the corresponding observation-model parameters and validating them against measurements from low-observable UAV platforms.

## 7. Conclusions and Future Works

The paper presented EdgeTwin-DRL, an edge-assisted digital twin framework for counter-drone detection and response optimization using cooperative multi-agent deep reinforcement learning. Rather than proposing a new reinforcement-learning algorithm, the framework integrates heterogeneous sensing, synchronized digital twin state management, edge-resource allocation, cooperative decision-making, and candidate-response evaluation within a unified counter-UAS decision architecture. The digital twin maintains a persistent representation of the protected airspace using radar, EO/IR, RF, and acoustic observations, while the MAPPO-based controller uses this synchronized state to coordinate computational allocation, detection sensitivity, and response recommendations at the edge.

The simulation-based evaluation across modeled urban, suburban, and open-field conditions showed that EdgeTwin-DRL provides favorable comparative performance relative to the evaluated cloud-centric, independent-learning, rule-based, and multi-agent baselines. Within the calibrated simulation environment, the framework achieved a detection accuracy of 97.3%, an F1-score of 95.8%, a false-positive rate of 1.4%, a mean detection-to-response latency of 527 ms, and a simulated response-success rate of 92.1%. Under the communication and computational assumptions adopted in the simulator, the mean latency was reduced by 72% relative to Cloud-DRL and by 26% relative to the MAPPO configuration without calibrated, environment-dependent sensing. These findings support the feasibility of combining synchronized airspace-state representation, edge processing, and cooperative decision-making for low-latency counter-UAS decision support under the modeled conditions.

The present results, however, establish simulation-level feasibility and comparative performance rather than operational field performance. The RF sensing models are calibrated and independently validated using publicly available datasets, while the remaining sensing components are parameterized using published experimental measurements; these sources improve the empirical grounding of the simulated observation models but do not constitute end-to-end validation of EdgeTwin-DRL. Physical deployment may introduce sensor–model mismatch, calibration drift, unmodeled clutter and interference, communication jitter, hardware-specific processing delays, environmental variability, and threat behaviors that are not fully represented in the current simulation.

Future work will therefore prioritize a staged simulation-to-real validation process. The first stage will involve hardware-in-the-loop evaluation using physical edge devices and sensing components to characterize inference latency, synchronization behavior, resource consumption, and sensor–model mismatch. This will be followed by controlled, passive detection-only field trials and site-specific recalibration before any active-response evaluation is considered. Future deployment studies should also incorporate explicit fail-safe mechanisms for stale or inconsistent digital twin states, uncertainty-aware response gating, and human authorization of active countermeasures within the applicable legal and regulatory framework. Additional research will investigate scalable multi-agent architectures for larger protected areas and UAV swarms, robustness against adversarial and sensor-spoofing attacks, recurrent or belief-state decision models for partial observability and non-Markovian effects, online adaptation to evolving threat behaviors, and integration with Unmanned Traffic Management (UTM) infrastructures. Physics-informed or hybrid physical-learning models will also be investigated to improve the physical consistency and interpretability of digital twin state prediction. Finally, federated multi-site learning and trustworthy digital twin synchronization will be explored to support the collaborative protection of geographically distributed critical infrastructures without requiring the exchange of raw sensing observations.

Future work may also investigate physics-informed or hybrid physical–learning models that incorporate UAV kinematic and sensor-domain constraints into digital twin state prediction to improve physical consistency, generalization, and interpretability under conditions that differ from those represented in the present simulation.

Further, a broader algorithmic benchmark incorporating alternative cooperative MARL approaches, including HAPPO and continuous-action variants of other multi-agent methods, would be valuable for determining whether the observed architectural benefits remain consistent across different learning algorithms.

Overall, our EdgeTwin-DRL provides a simulation-evaluated architectural foundation for investigating how edge-assisted digital twins and cooperative multi-agent learning can support responsive counter-UAS sensing and decision-making. Establishing its operational effectiveness will require the staged hardware and field validation described above.

## Figures and Tables

**Table 1:** Comparative summary of related work.

Ref.	Approach	DT	Edge	DRL	Multi-Agent	Joint Detection and Response
[5]	Radar counter-UAS survey	✗	✗	✗	✗	**✗**
[17]	RF + acoustic fusion DNN	✗	✗	✗	✗	**✗**
[18]	CNN sensor fusion (radar + camera)	✗	✗	✗	✗	**✗**
[13]	DRL drone interception (3D)	✗	✗	✓	✗	**✗**
[4]	Explainable DRL counter-drone	✗	✗	✓	✗	**✗**
[20]	DT cyber-attack detection (CPS)	✓	✗	✗	✗	**✗**
[22]	TwinSec-IDS (SDN + DT)	✓	✗	✗	✗	**✗**
[23]	TwinPort (DT + drone data)	✓	✗	✗	✗	**✗**
[28]	MADRL task offloading (UAV-MEC)	✗	✓	✓	✓	**✗**
[30]	DT-assisted offloading (vehicular)	✓	✓	✓	✗	**✗**
Ours	EdgeTwin-DRL	**✓**	**✓**	**✓**	**✓**	**✓**

DT = Digital Twin; DRL = Deep Reinforcement Learning; ✓ = supported; ✗ = not supported.

**Table 2:** Digital twin simulation parameters and domain randomization ranges.

Parameter	Value/Range	Randomization
Airspace dimensions	5 km × 5 km × 0.5 km	Fixed
Decision step frequency	10 Hz	Fixed
Episode duration	300 s (3000 steps)	Fixed
Number of drones (Cat. I)	1–2	Uniform
Number of drones (Cat. II)	1–3	Uniform
Number of drones (Cat. III)	3–5	Uniform
Drone speed	10–80 km/h	±15%
Drone RCS	0.001–0.1 m^2^	±25%
Radar detection range	200–5000 m	±15%
EO/IR detection range	100–2000 m	±20%
RF detection range	50–3000 m	±15%
Acoustic detection range	10–300 m	±20%
Sensor noise levels	SNR 5–30 dB	±20%
Wind speed	0–40 km/h	Uniform
Visibility	500 m–10 km	Uniform
Precipitation	None/Light/Heavy	Categorical
Time of day	Day/Dusk/Night	Categorical
Clutter density (radar)	Low/Medium/High	±30%
Edge inter-node latency	0.3 ± 0.1 ms (log-normal)	Stochastic
Cloud round-trip latency	50–200 ms (uniform)	Stochastic

**Table 3:** Training hyperparameters for EdgeTwin-DRL and DRL baselines.

Hyperparameter	Value
Total training steps	5 × 10^6^
Discount factor (γ)	0.99
GAE parameter (λ)	0.95
PPO clipping parameter (ε)	0.2
Learning rate (actor and critic)	3 × 10^−4^ (Adam)
Batch size	4096 transitions
PPO epochs per update	15
Mini-batch size	512 transitions
Actor hidden layers	[256, 128, 64] FC + ReLU + LayerNorm
Critic hidden layers	[256, 128, 64] FC + ReLU + LayerNorm
Entropy coefficient (c_2_)	0.01
Value loss coefficient (c_1_)	0.5
Max gradient norm	0.5
Number of agents (n)	4
Episode length	3000 steps (300 s simulated)
Parallel environments	64
Reward weights (w_1_, w_2_, w_3_, w_4_)	1.0, 0.8, 0.1, 0.3
Engagement threshold (τ_eng)	0.7
Random seeds	5 (0, 42, 123, 456, 789)

**Table 4:** Overall performance comparison (mean ± std across five seeds and 500 episodes). The best results are shown in bold.

Method	DA (%)	F1 (%)	FPR (%)	RSR (%)	Lat. (ms)	GPU (%)	$R_{cum}$
RB-Expert	87.2 ± 1.3	81.5 ± 1.8	6.8 ± 0.9	72.4 ± 2.1	1417 ± 95	25.0 ± 0.0	**124 ± 12**
Cloud-DRL	91.8 ± 1.5	87.3 ± 1.9	4.2 ± 0.7	78.1 ± 2.4	1910 ± 234	22.0 ± 1.2	**138 ± 18**
IPPO	93.1 ± 1.2	89.6 ± 1.5	3.5 ± 0.6	81.3 ± 2.0	970 ± 115	58.3 ± 3.1	**148 ± 15**
MADDPG	94.5 ± 1.0	91.2 ± 1.3	2.8 ± 0.5	84.7 ± 1.8	880 ± 102	52.1 ± 2.8	**176 ± 14**
MAPPO-NoCalib	95.8 ± 0.8	93.1 ± 1.1	2.1 ± 0.4	87.5 ± 1.5	713 ± 75	45.6 ± 2.4	**221 ± 16**
EdgeTwin-DRL	97.3 ± 0.6	95.8 ± 0.8	1.4 ± 0.3	92.1 ± 1.2	527 ± 52	41.2 ± 2.0	283 ± 13

DA = Detection Accuracy; F1 = F1-Score; FPR = False-Positive Rate (↓); RSR = Response Success Rate; Lat. = Latency (↓); GPU = GPU (↓); $R_{cum}$ = Cumulative Reward.

## Data Availability

The data presented in this study are available on request from the corresponding author due to restrictions.
